# Morphological and Molecular Characterization of a New *Isospora* (Apicomplexa: Eimeriidae) Species From a Singing Honeyeater (*Gavicalis virescens* Vieillot, 1817) (Passeriformes: Meliphagidae) in Western Australia

**DOI:** 10.1002/ece3.70801

**Published:** 2025-01-19

**Authors:** Yinhua Chen, Belinda Brice, Bruno P. Berto, Rongchang Yang

**Affiliations:** ^1^ Department of Resources and Environmental Sciences Maotai College Renhuai Guizhou China; ^2^ Kanyana Wildlife Rehabilitation Centre Lesmurdie Western Australia Australia; ^3^ Departamento de Biologia Animal, Instituto de Ciências Biológicas e da Saúde Universidade Federal Rural do Rio de Janeiro Seropédica Rio de Janeiro Brazil; ^4^ School of Agricultural Sciences Murdoch University Murdoch Western Australia Australia

**Keywords:** 18S rRNA, 28S rRNA, Coccidia, COI gene, *Isospora*, Singing honeyeater

## Abstract

We describe and characterize a new *Isospora* species from the Singing honeyeater (*Gavicalis virescens*) in Western Australia, both morphologically and molecularly. Microscopic analysis of a fecal sample identified 25 ellipsoidal oocysts, measuring 21–25 × 18–20 μm (mean 23.4 × 18.7 μm), with a length/width (L/W) ratio of 1.2–1.3 (mean 1.25). The oocyst wall is bilayered and ~1.0 μm thick, with a smooth outer layer making up about two‐thirds of the thickness. A micropyle cap, measuring ~2.0 × 4.5 μm, is present as a curved protrusion on the outer layer. The micropyle itself is ~4.5 μm wide. The oocysts lack a residuum but contain 1–2 polar granules. The 25 ellipsoidal sporocysts measured 14–15 × 8–9 μm (mean 14.1 × 8.7 μm) with an L/W ratio of 1.6–1.7 (mean 1.62). The Stieda body is flattened (~0.5 × 1.5 μm), the sub‐Stieda body is rounded (~1.5 × 2.5 μm), and the para‐Stieda body is absent. The sporocyst residuum, composed of clustered spherules, is ~3.5 × 3.5 μm. Sporozoites contain anterior and posterior refractile bodies and a nucleus. Molecular analysis of the 18S rRNA, 28S rRNA, and COI gene loci showed a 99.5% genetic similarity to *Isospora neochmiae* at the 18S rRNA locus and 99.2% and 97.7% similarities to *Isospora manorinae* at the 28S rRNA and COI loci, respectively. Phylogenetic analysis confirmed that the new species is closely related to *I. manorinae*. Based on these data, we propose this isolate as a new species, *Isospora virescensae* n. sp.

## Graphical abstract

Description of *Isospora virescensae* n. sp. from a Singing honeyeater *Gavicalis virescens*. Morphology study: most close to *I. manorinae*. Genetic study: 99.5% similar to *I. neochmiae* at 18S locus, 99.2% and 97.7% similar to *I. manorinae* at 28S and COI loci, respectively.

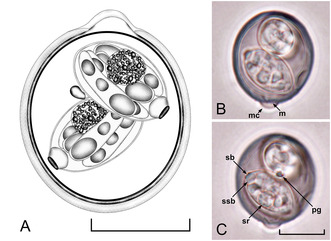

## 1 Introduction

The Singing honeyeater (*Gavicalis virescens*) is a native Australian bird, which is widely distributed across the Australian continent, except in the eastern coastal regions. It is mainly olive‐brown with a pale gray underbelly, and is also known as the Gray Peter or Grape‐eater (Pizzey and Knight 2007). It is a medium‐sized bird belonging to the order Passeriformes, family Meliphagidae, and genus *Gavicalis* (Pizzey and Knight 2007). It typically inhabits thickets along rivers and streams, vineyards, orchards, gardens, open shrublands, and mallee scrubs, feeding on fruits, berries, small insects, and grubs. There are 72 species of Australian honeyeaters and another 110 that are found predominantly in New Zealand and Papua New Guinea (Pizzey and Knight 2007).

Coccidia are unicellular protozoan parasites, mostly host‐specific, belonging to the phylum Apicomplexa and family Eimeriidae. They infect both invertebrates and vertebrates (Fayer 1980). The genus *Isospora* is the most common coccidia infecting passerine birds (Duszynski, Upton, and Couch 1999), yet there are limited genetic data available (Olson et al. 1998; Carreno and Barta 1999). The Kanyana Wildlife Rehabilitation Centre (KWRC), Perth, admits many wild birds and other wildlife needing treatment or rehabilitation into their facility each year. Many of these animals have their feces screened for intestinal parasites, including coccidia. Screening fecal samples for parasites and treating the parasitic infection, when necessary, can improve release rates of wildlife and reduce the amount of time the animals are kept in care. In Australia, *Isospora* species from honeyeaters that have been both morphologically and genetically characterized include *Isospora lesouefi* from captive‐bred Regent honeyeaters ( *Xanthomyza phrygia* ) (Morin‐Adeline et al. 2011), *Isospora phylidonyrisae* from a New Holland honeyeater ( *Phylidonyris novaehollandiae* ) (Yang, Brice, Berto, and Ryan 2021), *Isospora anthochaerae* from the Red wattlebird ( *Anthochaera carunculata* ) (Yang, Brice, and Ryan 2014), *Isospora lunulatae* from the Western wattlebird ( *Anthochaera lunulata* ) (Yang, Brice, Berto, and Zahedi 2021), and *Isospora manorinae* from a Yellow‐throated miner ( *Manorina flavigula wayensis* ) (Yang et al. 2016) in Western Australia (WA). Species that have only been morphologically described include *Isospora samoaensis* from the Wattled honeyeater ( *Foulehaio carunculatus* ) in American Samoa (Adamczyk, McQuistion, and LaPointe 2004). Other morphologically and genetically characterized *Isospora* species in WA include *Isospora streperae* from a Gray currawong ( *Strepera versicolor plumbea* ) (Yang, Brice, Al Habsi, et al. 2015) and *Isospora neochmiae* from a captive‐bred Red‐browed finch ( *Neochmia temporalis* ) (Yang, Brice, and Ryan 2015). The genetic characterization of these *Isospora* species in WA was conducted using the 18S rRNA, 28S rRNA, and COI loci (Yang, Brice, and Ryan 2014, 2015; Yang, Brice, Al Habsi, et al. 2015; Yang et al. 2016; Yang, Brice, Berto, and Ryan 2021). For *I. lesouefi*, characterization was based solely on the partial COI gene sequence (Morin‐Adeline et al. 2011).

This study describes the morphological and genetic characteristics of a new *Isospora* species infecting the Singing honeyeater in WA, proposing the species name *Isospora virescensae* n. sp., reflecting its host, the Singing honeyeater (*G. virescens*).

## 2 Materials and Methods

### 2.1 Sample Collection and Storage

A wild adult Singing honeyeater was admitted to the KWRC, Perth, in April 2014 after it was found on the ground by a member of the public. Medical assessment revealed that the bird had ataxia, and the right leg appeared weak. It was otherwise in good body condition. Treatment was implemented but the bird died the following day. A fecal sample was collected shortly after admission and stored at 4°C in a labeled vial until parasitological examination was performed. All procedures were approved and monitored by the Murdoch University Animal Ethics Committee (approval number R2352/10).

### 2.2 Morphological Analysis

Preliminary parasitological examination (wet mount and fecal float) of the fecal sample was conducted at KWRC. A saturated sodium chloride and 50% sucrose (w/v) solution were used for the fecal float. Numerous unsporulated coccidian oocysts were observed by microscopy. The fecal sample was emulsified in a 2% (w/v) potassium dichromate solution (K_2_Cr_2_O_7_) and stored at 4°C until transport to Murdoch University (within 24 h). Upon arrival at Murdoch University, the dichromate/oocyst suspension was poured into a thin layer at the bottom of a Petri dish. The Petri dish was stored at room temperature (20°C–22°C), in the dark, to encourage sporulation of the oocysts. An Olympus CH‐2 binocular microscope was used to regularly screen the sample for oocyst sporulation and to observe the morphological characteristics of the sporulated oocysts.

### 2.3 DNA Extraction

DNA was extracted from 200 mg of each fecal sample, which was from the same bird at three different time points, using a Power Soil DNA Kit (MolBio, Carlsbad, CA), with modifications based on Yang et al. (2023). Briefly, samples underwent four cycles of freeze/thaw using liquid nitrogen and boiling water to effectively lyse oocysts before following the manufacturer's protocol. A negative control (no fecal sample) was also included.

### 2.4 PCR Amplification and Sequence Analysis

Partial fragments of the 18S rRNA were obtained by a PCR with the protocol described by Eberhard et al. (1999); the primers (CRYPTOF 5′‐AAC CTG GTT GAT CCT GCC AGT and CRYPTOR 5′‐GCT TGA TCC TTC TGC AGG TTC ACC TAC) were used, and the PCR reaction contained 2.5 μL of 10 × Kapa PCR buffer, 3 μL of 25 mM MgCl_2_, 1.5 μL of 10 nM dNTPs, 10 nM each of primer, 1 unit of KapaTaq (Geneworks, Adelaide, SA), 1 μL of DNA (~50 ng), and 14.9 μL of H_2_O. PCR cycling conditions were 1 cycle of 94°C for 3 min, followed by 45 cycles of 94°C for 30 s, 55°C for 30 s, and 72°C for 2 min and a final extension of 72°C for 5 min. 28S rRNA and COI genes were amplified through nested PCRs using the primers and PCR conditions described by Yang et al. (2023). The nested PCR approach, involving two successive rounds of amplification with distinct primer sets, enhances the specificity and yield of the target gene fragments. Following amplification, the PCR products for all three genetic loci were purified. This purification was carried out using an in‐lab‐developed quick method, ensuring the removal of contaminants efficiently and effectively (Yang et al. 2013). The purified PCR products were then subjected to bidirectional sequencing to achieve high‐quality, reliable sequence data. This sequencing was conducted using an ABI Prism Dye Terminator Cycle Sequencing kit (Applied Biosystems, Foster City, CA, USA), strictly following the manufacturer's protocols as outlined by Yang et al. (2016).

### 2.5 Phylogenetic Analysis

The 18S rRNA, 28S rRNA, and COI gene sequences from *I. virescensae* n. sp. were aligned with other closely related *Isospora* and *Eimeria* sequences retrieved from GenBank (Benson et al. 2005) via BLAST (Altschul et al. 1990) searches. Phylogenetic trees of the three gene sequences were constructed using maximum likelihood method in MEGA‐X (Kumar et al. 2018) with the most appropriate nucleotide substitution models (TN93 + G + I, TN93 + G + I, and GTR + G + I, respectively).

Three identical 1107 bp 18S rRNA sequences of *I. virescensae* n. sp. were obtained from the oocysts in the fecal samples of the Singing honeyeater ( *G. virescens* ). These sequences were aligned with 26 other *Isospora* spp. sequences, 14 *Eimeria* spp., and 1 *Caryospora* sp. The reference sequences were selected based on NCBI Blast similarities (one sequence per species) and included all available sequences for *Isospora* spp. in birds. A sequence of *Toxoplasma gondii* (Nicolle & Manceaux, 1908) (L24381) was used as the outgroup. Three identical 28S rRNA sequences (1367 bp) from the oocysts in the fecal samples of the Singing honeyeater ( *G. virescens* ) were aligned with 41 sequences from other *Isospora* species from birds and one sequence from *Eimeria* sp. (*Eimeria papillata*). Similarly, for the 18S rDNA gene, the selection of the 28S rRNA reference sequences was based on NCBI Blast similarities and covered all *Isospora* species sequences. *T. gondii* was used as the outgroup (L25635).

A 633‐bp unique partial COI gene sequence of *I. virescensae* n. sp. was compared with 25 sequences from other *Isospora* spp. and one *Caryospora* sp. sequence. *T. gondii* was used as the outgroup.

### 2.6 Line Drawing

Line drawings were edited using two software applications from CorelDRAW (Corel Draw Graphics Suite, Version 2020; Corel Corporation, Canada), i.e., Corel DRAW and Corel PHOTO‐PAINT (Yang, Brice, Berto, and Ryan 2021).

## 3 Results

### 3.1 Morphological Analysis

#### 3.1.1 Species Description

**FIGURE 1:**
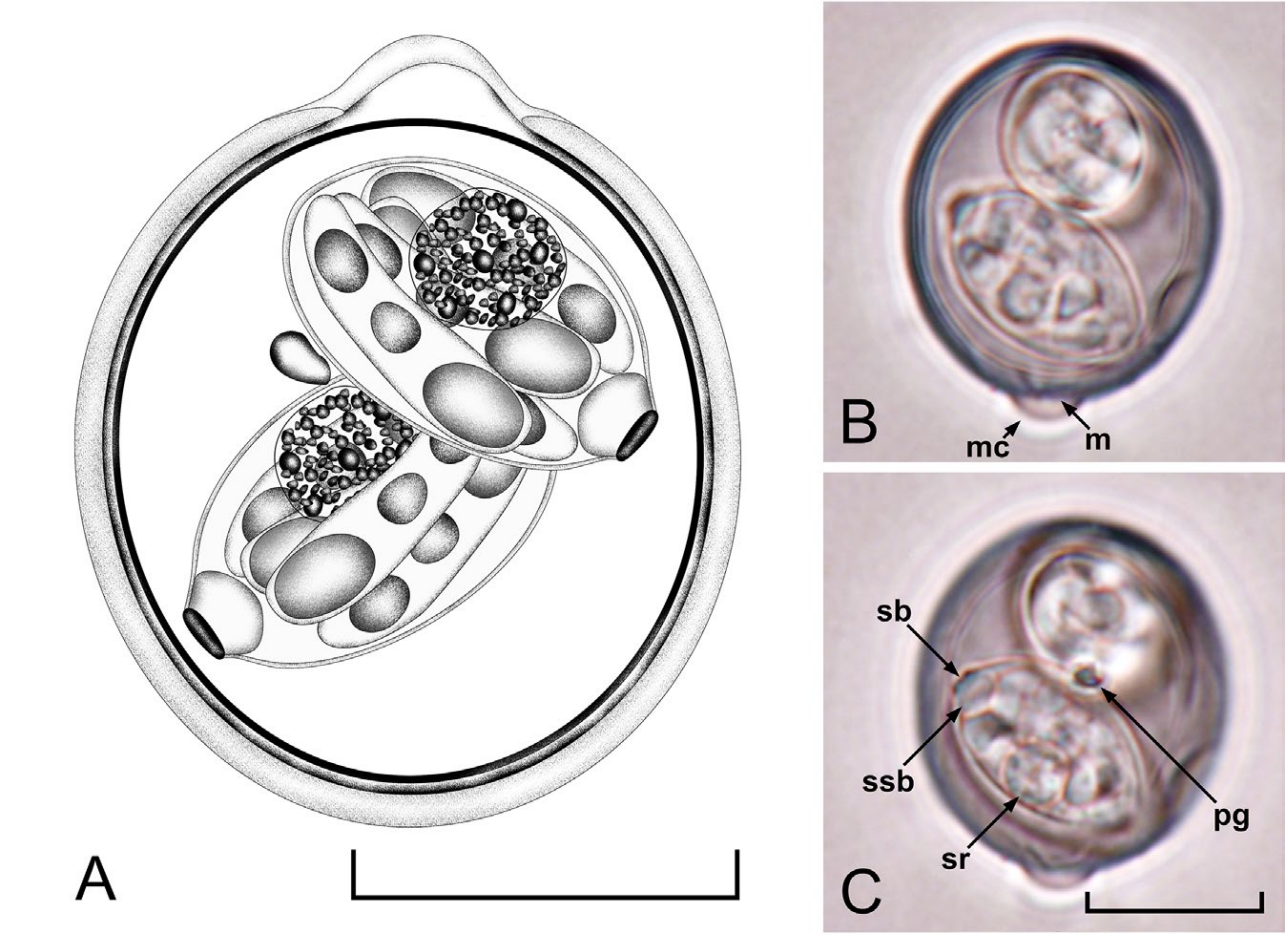
Composite line drawing and photomicrographs of sporulated oocysts of *I. virescensae* n. sp. from a Singing honeyeater (*Gavicalis virescens*) in Western Australia. Note the micropyle (m), micropyle cap (mc), polar granule (pg), sporocyst residuum (sr), and Stieda (sb) and sub‐Stieda (ssb) bodies. Scale bars: 10 μm.

Oocysts (*n* = 25) ellipsoidal, 21–25 × 18–20 (23.4 × 18.7); length/width (L/W) ratio 1.2–1.3 (1.25). Wall bilayered, ~1.0 thick, outer layer smooth, c. 2/3 of total thickness. Micropyle cap present as a curved protrusion on the outer layer of the oocyst wall, ~2.0 × 4.5. Micropyle present with no invagination of the inner layer, ~4.5 wide. Oocyst residuum absent, but one to two polar granules are present. Sporocysts (*n* = 25) ellipsoidal, 14–15 × 8–9 (14.1 × 8.7); L/W ratio 1.6–1.7 (1.62). Stieda body present, flattened, ~0.5 × 1.5; sub‐Stieda body rounded, ~1.5 × 2.5; and para‐Stieda body absent; sporocyst residuum present, composed of some spherules clustered by a membrane, in a rounded shape, ~3.5 × 3.5. Sporozoites with anterior and posterior refractile bodies and nucleus (Figure 1).

Host: Singing honeyeater (*Gavicalis virescens* Vieillot, 1817).

Locality: Dalkeith (−31°59′49.20″ S, 115°47′49.20″ E., Perth, Western Australia).

Prevalence: Unknown.

Other hosts: Unknown.

Prepatent period: Unknown.

Patent period: Unknown.

Site of infection: Unknown, oocysts collected from feces.

Sporulation time: 48–72 h.

Material deposited: DNA sequences have been deposited in GenBank under accession numbers PQ108887, PQ108888, and PQ110232 for the 18S, 28S, and COI loci, respectively.

### 3.2 Phylogenetic Analysis

#### 3.2.1 18S rRNA

**FIGURE 2:**
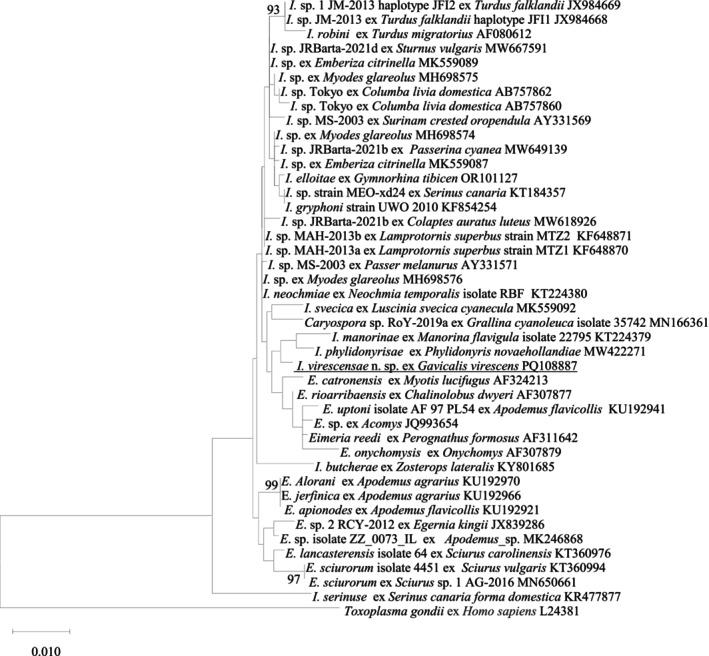
Evolutionary relationships of *I. virescensae* n. sp. inferred by maximum likelihood (ML) analysis of 18S rDNA sequences (1107 bp). Percentage support (> 70%) from 1000 pseudoreplicates from the ML analysis is indicated at the left of the support nodes.

*I. virescensae* n. sp. shared a 99.5% similarity with *I. neochmiae* (KT224380) and *I. elliotae* (OR101127) based on a pairwise comparison. As shown in Figure 2, *I. virescensae* n. sp. grouped with two *Isospora* spp. from WA: *I. manorinae* (KT224379) and *I. phylidonyrisae* (MW422271). When comparing the genetic distances between *I. virescensae* n. sp. and 42 reference species/isolates, the shortest pairwise genetic distance was observed with *Isospora* sp. ex *Myodes glareolus* (MH698574) at 0.0045. In contrast, some reference 18S rRNA sequences showed even smaller genetic distance values. For instance, *Isospora* sp. ex *Myodes glareolus* (MH698576) had a genetic distance of “0” with *I. neochmiae* ex *Neochmia temporalis* isolate RBF (KT224380) from Australia, as well as with *Isospora* sp. MAH‐2013b ex *Lamprotornis superbus* strain MTZ2 (KF648871) and *Isospora* sp. MAH‐2013a ex *Lamprotornis superbus* strain MTZ1 (KF648870) from Canada (Table S1).

#### 3.2.2 28S rRNA

**FIGURE 3:**
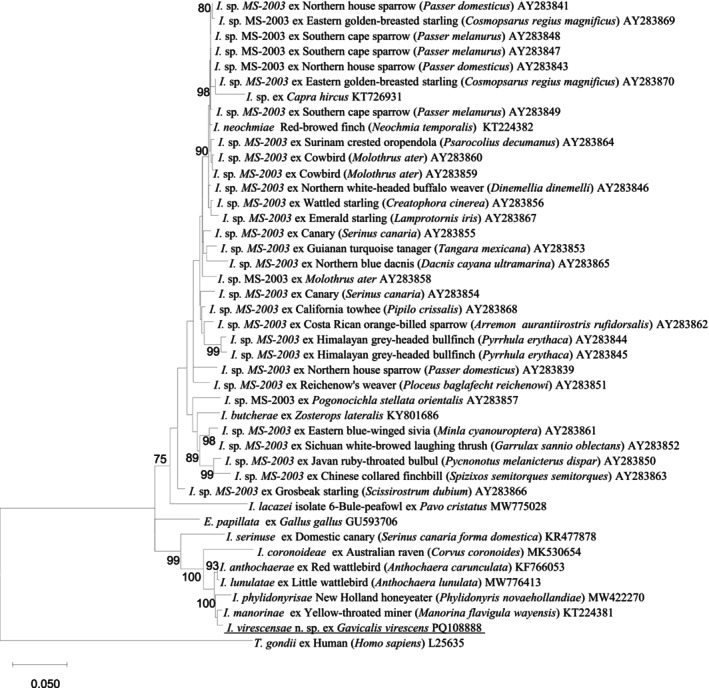
Evolutionary relationships of *I. virescensae* n. sp. inferred by maximum likelihood (ML) analysis of 28S rDNA sequences (1367 bp). Percentage support (> 70%) from 1000 pseudoreplicates from the ML analysis is indicated at the left of the nodes.

Phylogenetic analysis showed that *I. virescensae* n. sp. was most close to *I. manorinae* (KT224381) from a Yellow‐throated miner ( *M. flavigula obscura* ) with a genetic similarity of 99.0% and *I. phylidonyrisae* (MW422270) from a New Holland honeyeater ( *P. novaehollandiae* ) with a genetic similarity of 99.2%, forming a separate clade. As shown in Figure 3, *I. anthochaerae* (KF766053), *I. coronoideae* (MK530654), *I. serinuse* (KR477878), and *I. lunulatae* (MW776413), along with the three *Isospora* species mentioned above (including the new species *I. virescensae* n. sp.), formed a strongly supported clade in the phylogenetic tree. All seven *Isospora* species were identified from passerine birds in WA. The comparison of genetic distances between *I. virescensae* n. sp. and 42 reference sequences revealed that the shortest distance was with *I. manorinae* ex *Manorina flavigula wayensis* (KT224381) at 0.008346. However, some genetic distances among the 28S rRNA reference sequences were smaller than this value (Table S2).

#### 3.2.3 COI Gene

**FIGURE 4:**
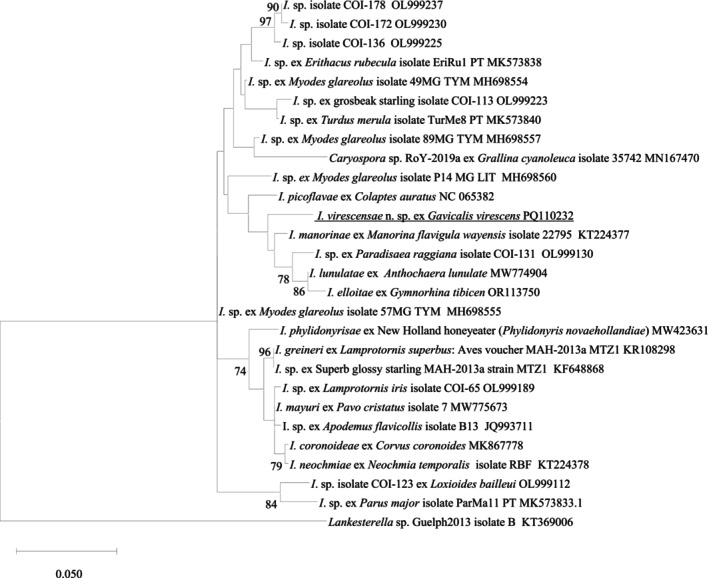
Evolutionary relationships of *I. virescensae* n. sp. inferred by maximum likelihood (ML) analysis of partial *cox*1 gene sequences (633 bp). Percentage support (> 70%) from 1000 pseudoreplicates from the ML analysis is indicated at the left of the nodes.

In the phylogenetic tree, a similar pattern to Figure 3 was observed, showing that *I. virescensae* n. sp. is most closely related to *I. manorinae* (KT224381), with the highest genetic similarity of 97.7%, along with *I. lunulatae* (MW774904) and *I. elloitae* (OR113750) as well as an unnamed *Isospora* spp. (OL999130), forming a clade (Figure 4). It was shown that the shortest genetic distance between *I. virescensae* n. sp. and the reference COI gene sequences was 0.022644, with *I. manorinae* (KT224381). This value is larger than some of the distances observed between the COI reference sequences (Table S3).

## 4 Discussion

Sporulated oocysts of *I. virescensae* n. sp. are morphologically different from other previously characterized *Isospora* spp. recorded from Artamidae or Passeriformes of Oceania (Trachta, Evandro, and Literák 2006; Yang, Brice, and Ryan 2014, 2015; Yang, Brice, Al Habsi, et al. 2015; Yang, Brice, Elliot, et al. 2015; Yang et al. 2016; Yang, Brice, Berto, and Ryan 2021; Yang, Brice, Berto, and Zahedi 2021; Yang et al. 2023). As shown in Table 1, the oocyst dimensions of *I. virescensae* n. sp. (23.4 × 18.7 μm) are in a similar size range as those of *I. manorinae* (22.8 × 18.3 μm), which was identified from a Yellow‐throated miner ( *M. flavigula wayensis* ) (Yang et al. 2016) (Table 1). The oocyst of *I. virescensae* n. sp. is ellipsoidal in shape with an L/W ratio of 1.25, whereas the oocyst of *I*. *manorinae* is spherical to subspherical in shape with an L/W ratio of 1.2. Another significant difference between *I. virescensae* n. sp. and *I. manorinae* is the micropyle, which is present in *I. virescensae* n. sp. without invagination of the inner layer and is ~4.5 μm wide, while the micropyle is absent in *I. manorinae*.

**TABLE 1:** Morphological comparison of *I. virescensae* n. sp. with other *Isospora* species in the passerine birds.

Coccidia	Hosts	References	Oocysts						Sporocysts				
			Shape	Measurements (μm)	Shape index	Wall (μm)	Polar granule	Oocyst residuum	Shape	Measurements	Stieda body	Sub‐Stieda body	Residuum
*I. anthochaerae*	*Anthochaera carunculata*	Yang, Brice, and Ryan (2014)	Subspherical	23.4 × 20.7 (20.0–26.0 × 19.0–22.0)	1.1	Bi‐layered c. 0.8	−	−	O	14.5 × 10.1 (11.0–17.0 × 9.0–11.0)	Hemi‐dome	Rectangular‐shaped	Compact
*I. braziliensis*	*Oryzoborus angolensis*	Trachta, Evandro, and Literák (2006)	Spherical to subspherical	17.8 × 16.9 (16–19 × 16–18)	1	One‐layered c. 1.0	−	−	E	13.2 × 10.8 (12–14 × 9–12)	Tiny	Absent	Scattered granules
*I. canaria*	*Serinus canaria* Linnaeus	Box (1975), Berto et al. (2013)	Subspherical to ellipsoidal	24.6 × 21.8 (17–30 × 17–30)	1.1	Tri‐layered c. 1.2	+	−	Lemon	18.1 × 11.5 (17.0–22.0 × 1.00–13.0)	Nipple‐like	2.0 × 3.0	Compact
*I. curio*	*Oryzoborus angolensis*	Trachta, Evandro, and Literák (2006)	Spherical to subspherical	24.6 × 23.6 (22–26 × 22–25)	1	Bilayerd c. 1.5	−	−	O	13.2 × 10.9 (15–17 × 10–13)	Small	Absent	Scattered granules
*I. daphnensis*	*Geospiza fortis*	McQuistion (1990)	Ellipsoidal	27.3 × 23.6 (22–30 × 20–27)	1.2	Bi‐layered c. 1.5	+	−	O	15.2 × 10.2 (15.0–16.0 × 9.0–11.0)	Nipple‐like	Small	Scattered granules
*I. elliotae*	*Gymnorhina tibicen*	Yang et al. (2023)	Subspherical	20.7 × 18.7 (19.8–21.6 × 18–19.6)	1.1	Bi‐layered c. 1.5	+	−	O	12.6 × 9.7 (11.9–13.2 × 8.9–10.8)	Flattened to half‐moon	Indistinct	Compact
*I. exigua*	*Camarhynchus parvulus*	McQuistion and Wilson (1988)	Subspheroidal	20.4 × 20.1 (20–23 × 18–23)	1	One‐layered	−	−	O	14 × 9.5 (13–15 × 8–10)	Small	Small	Irregular‐shaped
*I. fragmenta*	*Camarhynchus parvulus*	McQuistion and Wilson (1988)	Subspheroidal	25.3 × 24.2 (24–27 × 23–25)	1	One‐layered	+	−	Piriform	15.4 × 11.5 (14–17 × 11–12)	Knob‐like	Prominent	Irregular‐shaped
*I. gryphoni*	*Carduelis tristis* Linnaeus	Olson et al. (1998)	Spherical	29.2 × 30.7 (25.0–33.0 × 28.0–34.0)	1	Bi‐layered c. 0.8	+	−	O	22.2 × 13.4 (15–25.0 × 12.0–14.5)	Small	Indistinct	Prominent
*I. lunulatae*	*Anthochaera lunulata*	Yang, Brice, Berto, and Zahedi (2021)	Subspheroidal	30.6 × 29.4 (27–34 × 26–31)	1.04	Bi‐layered c. 1.0	+	−	O	18.3 × 10.7 (17–19 × 10–12)	Flattened to rounded	Rounded to rectangular	Compact
*I. manorinae*	*Manorina flavigula obscura*	Yang et al. (2016)	Spherical to subspherical	22.8 × 18.3 (20.3–23.8 × 17.7–18.7)	1.2	Bi‐layered c. 1.3	+	−	Lemon	15.5 × 9.7 (14.6–15.7 × 9.5–9.7)	Hemi‐dome	Rectangular‐shaped	Compact
*I. neochmiae*	*Neochmia temporalis*	Yang, Brice, and Ryan (2015)	Spherical	18.3 × 18.2 (18.2–18.9 × 18.2–18.6)	1	Bi‐layered c. 1.2	+	−	O	13.3 × 8.6 (9.5–16.4 × 6.8–10.0)	Indistinct	Absent	Compact
*I. paranaensis*	*Oryzoborus angolensis*	Trachta, Evandro, and Literák (2006)	Subspherical to broadly ellipsoid	24.3 × 19.8 (22–26 × 18–22)	1.2	One‐layered c. 1.5	+	−	O	15.7 × 10.1 (14–18 × 8–12)	Distinct	Distinct	Spherical
*I. phylidonyrisae*	*Phylidonyris novaehollandiae*	Yang, Brice, Berto, and Ryan (2021)	Subspheroidal	29.8 × 29.4 (29–32 × 28–31)	1.01	Bi‐layered c. 1.5	+	−	O	18.4 × 12.3 (18–19 × 12–14)	Flatted	Rounded	Scattered granules
*I. rotunda*	*Camarhynchus parvulus*	McQuistion and Wilson (1988)	Subspheroidal	20.9 × 20.8 (20–24 × 19–23)	1	One‐layered	+	−	O	15 × 9.7 (13–16 × 9–10)	Knob‐like	Prominent	Round
*I. serini*	*Serinus canaria* Linnaeus	Box (1975), Speer and Duszynski (1975)	Spherical to subspherical	20.1 × 19.2 (13.0–23.0 × 13.0–23.0)	1	Tri‐layered c. 1.2	−	−	E	15.2 × 9.4 (13.0–16.0 × 8.0–11.0)	2.0 × 0.6	5.0 × 3.0	Scattered granules
*I. serinuse*	*Serinus canaria* forma *domestica*	Yang, Brice, Elliot, et al. (2015)	Spherical to subspherical	25.5 × 23.5 (24.4–27.0 × 22.0–24.8)	1.09	Bi‐layered c. 1.2	+	−	Lemon	18.9 × 11.8 (17.8–20.2 × 10.6–13.0)	Small	Indistinct	Compact
*I. streperae*	*Strepera versicolor*	Yang, Brice, Al Habsi, et al. (2015)	Spherical	23.8 × 22.5 (22–24.5 × 21.8 × 24.5)	1.06	Bi‐layered c. 1.0	−	+	O	14.4 × 11.2 (11.5–15.8 × (10.4–12.5)	Hemi‐dome	Rectangular‐shaped	Compact
*I. temeraria*	*Geospiza fortis*	McQuistion and Wilson (1988)	Subspheroidal	25.4 × 21.1 (21–30 × 17–23)	1.2	One‐layered	+	−	Piriform	15 × 10 (14–15 × 9–11)	Knob‐like	Prominent	Round
*I. tristum*	*Acridotheres tristis*	Madani et al. (2018)	Spherical to subspherical	23.3 × 22.3 (18.5–30 × 18.1–29.3)	1.05	Bi‐layered c. 1.3	−	−	O	13.9 × 9.3 (10.2–17.5 × 6.5–12.2)	Flatted	Rounded	Compact
*I. virescensae* n. sp.	*Gavicalis virescens*	This study	Ellipsoidal	23.4 × 18.7 (21–25 × 18–20)	1.25	Bi‐layered c.1.0	+	−	E	14.1 × 8.7 (14–15 × 8–9)	Flatted	Rounded	Compact

*Note:* − = absent; + = present.

Abbreviations: E = elipsoidal; O = ovoidal.

Interestingly, the Yellow‐throated miner (also a honeyeater) and the Singing honeyeater share similar home ranges. The Singing honeyeater is often seen foraging in the same area as the Red wattlebird and other honeyeaters in the Perth area. Each of these honeyeater species has their own specific *Isospora* species infecting them. We have previously described new *Isospora* species from the Red wattlebird ( *A. carunculata* ), a Yellow‐throated miner ( *M. flavigula wayensis* ), the Western wattlebird ( *A. lunulata* ), and from the New Holland honeyeater ( *P. novaehollandiae* ). The Singing honeyeater in this study was not displaying any signs of coccidiosis. Another seven Singing honeyeaters were sampled and screened for coccidian oocysts at KWRC over the 2014–2016 period. Of these seven Singing honeyeaters, oocysts were seen in two of these birds (2/7, 28.6%). These samples were not sequenced so it is impossible to say if they were *I. virescensae* n. sp. Molecular technology has played a significant role in organism taxonomy. This study demonstrates that genomic sequence data can be a complementary source for coccidian identification. For example, *I. virescensae* n. sp. was morphologically similar to the oocysts in *I. manorinae* except for the feature of the oocyst micropyle. However, the 18S rRNA sequence of *I. virescensae* n. sp. showed a 99.5% similarity with *I. neochmiae* and *I. elliotae*, while it exhibited the highest genetic similarities at the 28S rRNA and COI gene loci with *I. manorinae*, at 99.1% and 97.7%, respectively. This further confirms that *I. virescensae* n. sp. is most closely related to *I. manorinae*, but they are two distinct species. It also highlights the importance of using multiple loci for coccidian molecular identification. While the 18S rRNA locus is used most often in coccidian molecular taxonomy, its highly conserved nature necessitates the use of additional loci for accurate identification. The use of molecular data has revolutionized the identification and classification of parasitic organisms, providing a level of precision that morphological characteristics alone cannot achieve. This is particularly true for coccidians, where subtle morphological differences can be challenging to discern. The integration of molecular methods, such as sequencing of the 18S rRNA, 28S rRNA, and COI gene loci, allows for a more comprehensive understanding of phylogenetic relationships and species boundaries.

## Author Contributions

**Yinhua Chen:** formal analysis (equal), investigation (equal), methodology (equal), writing – original draft (equal). **Belinda Brice:** data curation (equal), investigation (equal), resources (equal), writing – original draft (equal). **Bruno P. Berto:** software (equal), validation (equal), writing – review and editing (equal). **Rongchang Yang:** conceptualization (lead), supervision (lead), writing – review and editing (equal).

## Conflicts of Interest

The authors declare no conflicts of interest.

## Supporting information


Table S1

Table S2

Table S3

## Data Availability

The 18S, 28S, and COI additional sequence data generated from this study are accessible at the public domain: https://www.ncbi.nlm.nih.gov/nuccore under the GenBank accession numbers of PQ108887, PQ108888, and PQ110232 for the 18S, 28S, and COI loci, respectively. The 18S, 28S, and COI additional sequence data used in the phylogenetic analysis were derived from the following resources available in the public domain: https://www.ncbi.nlm.nih.gov/nuccore with the GenBank accession numbers in Figures 2, 3, 4.
