# Correction to “Morphological and Molecular Characterization of a New *Isospora* (Apicomplexa: Eimeriidae) Species From a Singing Honeyeater (*Gavicalis virescens* Vieillot, 1817) (Passeriformes: Meliphagidae) in Western Australia”

**DOI:** 10.1002/ece3.71689

**Published:** 2025-07-01

Chen, Y., Brice, B., Berto, B.P. and Yang, R. (2025), Morphological and Molecular Characterization of a New *Isospora* (Apicomplexa: Eimeriidae) Species From a Singing Honeyeater (*Gavicalis virescens* Vieillot, 1817) (Passeriformes: Meliphagidae) in Western Australia. *Ecol Evol*, 15: e70801. https://doi.org/10.1002/ece3.70801

On the front page, the affiliation of the first author, Yinhua Chen, was listed as “Department of Resources and Environmental Sciences, Maotai College, Renhuai, Guizhou, China.” The correct affiliation should be “Moutai Institute, Renhuai, Guizhou, China.”

We apologize for this error.

